# L-Lactate protects neurons against excitotoxicity: implication of an ATP-mediated signaling cascade

**DOI:** 10.1038/srep21250

**Published:** 2016-02-19

**Authors:** P. Jourdain, I. Allaman, K. Rothenfusser, H. Fiumelli, P. Marquet, P. J. Magistretti

**Affiliations:** 1 Brain Mind Institute, Ecole Polytechnique Fédérale de Lausanne (EPFL), 1015 Lausanne, Switzerland; 2 King Abdullah University of Science and Technology (KAUST), Thuwal, Kingdom of Saudi Arabia; 3 Centre de Neurosciences Psychiatriques, CHUV, Département de Psychiatrie, Site de Cery, CH-1008 Prilly/Lausanne, Switzerland

## Abstract

Converging experimental data indicate a neuroprotective action of L-Lactate. Using Digital Holographic Microscopy, we observe that transient application of glutamate (100 μM; 2 min) elicits a NMDA-dependent death in 65% of mouse cortical neurons in culture. In the presence of L-Lactate (or Pyruvate), the percentage of neuronal death decreases to 32%. UK5099, a blocker of the Mitochondrial Pyruvate Carrier, fully prevents L-Lactate-mediated neuroprotection. In addition, L-Lactate-induced neuroprotection is not only inhibited by probenicid and carbenoxolone, two blockers of ATP channel pannexins, but also abolished by apyrase, an enzyme degrading ATP, suggesting that ATP produced by the Lactate/Pyruvate pathway is released to act on purinergic receptors in an autocrine/paracrine manner. Finally, pharmacological approaches support the involvement of the P2Y receptors associated to the PI3-kinase pathway, leading to activation of K_ATP_ channels. This set of results indicates that L-Lactate acts as a signalling molecule for neuroprotection against excitotoxicity through coordinated cellular pathways involving ATP production, release and activation of a P2Y/K_ATP_ cascade.

Excitotoxicity is a pathological process leading to neuronal damage and death triggered by excessive stimulation by glutamate of N-Methyl-D-Aspartate (NMDA) receptors^1^. This overstimulation of NMDA receptors causes an intracellular Ca^2+^ overload which triggers, in turn, several downstream neurotoxic cascades^2^. Acute brain pathologies such as stroke and spinal cord injury engage excitotoxic processes which are associated with the neuronal loss observed in these pathologies^3^,^4^; excitotoxicity has also been proposed as a component of certain progressive neurodegenerative diseases such as Alzheimer’s or Amyotrophic Lateral Sclerosis^5^,^6^. The massive activation of NMDA receptor being the source of excitotoxic processes, compounds interfering with NMDA receptors have been developed for the management of such pathological conditions and diseases. However clinical trials of NMDA receptors antagonists have failed due to lack of effectivity, undesired or even cytotoxic side effects^7^,^8^. In this context, identification of novel neuroprotective compounds that can block the deleterious biochemical cascades activated downstream of NMDA receptors is therefore of interest.

Overstimulation of glutamate receptors is accompanied by a disruption of ionic homeostasis, which is restored through ATP-dependent ion pumps^9^,^10^. During neuronal activity, astrocyte-derived L-Lactate acts as an energy substrate to meet the increased energy demands of neurons^11^,^12^. L-Lactate is reported to protect mouse brain against excitotoxic^13^,^14^ as well ischaemic damage^15^,^16^ both after intracerebroventricular and intravenous administration^17^. The mechanisms by which L-Lactate induces neuroprotection against excitotoxic and ischemic insults is still unknown. A natural hypothesis would be a pure metabolic mechanism by which L-Lactate rescues the cellular energy charge^18^ to maintain intracellular levels of ATP sufficient to ensure ATP-dependent ion pumps efficiency. Alternatively, L-Lactate-driven ATP production via the tricarboxylic acid cycle may induce neuroprotection through the activation of specific purinergic receptors such as those on hippocampal interneurons where ATP activated P2Y1 receptors promote synaptic inhibition of neuronal networks^19^. Finally, one could also consider that L-Lactate directly acts as signalling molecule through membrane receptors^20^,^21^,^22^.

In the present study we have undertaken a detailed characterization of the cellular mechanisms associated with L-Lactate neuroprotection in a model of glutamate-induced excitotoxicity in cortical primary neuronal cultures. Glutamate-induced neuronal death is monitored by quantitative phase Digital Holographic Microscopy (QP-DHM), a new and non-invasive imaging technique allowing the visualization of cell structure and dynamics, including various biological processes in relation to transmembrane water transport, with a nanometric axial sensitivity^23^. The observation that glutamate-induced excitotoxicity triggers as an early-stage component^24^ a rapid and acute swelling of cell bodies and dendrites (*i.e.* an early marker of excitotoxicity) subsequent to Na^+^,Cl^−^ and water inflows^1^,^25^, has made DHM a useful technique to detect early-stages of neuronal excitotoxic death^26^,^27^. Results presented here show that L-Lactate acts as a signaling molecule conferring neuroprotection against excitotoxic insults through a set of coordinated mechanisms based on an increase in ATP production and release and the subsequent activation of the P2Y receptors, mainly P2Y2, leading to an intracellular neuroprotective signaling pathway involving PI3 kinase and K_ATP_ channels.

## Materials and Methods

### Culture preparation

Experiments are conducted in accordance with the Swiss Federal Guidelines for Animal Experimentation and are approved by the Cantonal Veterinary Office for Animal Experimentation (Vaud, Switzerland). Primary cultures of cortical neurons are prepared from E17 OF1 mice embryos of either sex (Charles River Laboratories, L'Arbresle, France) as previously described^28^. Briefly, embryos are decapitated and brains removed and placed in a PBS-glucose solution. Cortices are removed under a dissecting microscope, minced in 2 mm^2^ pieces, and incubated at 37 °C for 30 min in a solution containing papain 20 U/ml (Worthington Biochemical, Lakewood, USA), 1 mM l-cysteine, 0.5 mM EDTA, and 100 U/ml DNAase (Worthington Biochemical, Lakewood, USA). Papain activity is then inhibited by adding fetal calf serum and a single-cell suspension is obtained by gentle trituration in Neurobasal medium supplemented with B27 and GlutaMAX (Invitrogen, Basel, Switzerland). Cells are plated at an average density of 15000 cells/cm^2^ in supplemented Neurobasal medium on poly-ornithine coated glass coverslips (20 mm ∅). Neurons were maintained at 37 °C in a humidified atmosphere of 95% air/5% CO_2_ and were used after 21–35 days *in vitro* (DIV). These culture conditions typically produced 93% pure neuronal cultures, as assessed by microtubule-associated protein 2 (MAP2) and glial fibrillary acidic protein (GFAP) co-immunostaining^29^.

### DHM imaging

QP-DHM is an interferometric imaging technique that allows to visualize transparent specimens, including living cells, by measuring the phase retardation induced by the specimens on a transmitted wave (for description of the basic design of the imaging system, see^30^). In the context of living cells recordings by QP-DHM, the phase retardation or the quantitative phase signal (QPS) is highly sensitive to the intracellular refractive index, which mainly depends on protein content^31^. Consequently, any transmembrane water movement, modifying the intracellular refractive index through a dilution or concentration process, drastically alter the QPS^31^. Therefore, a decrease of QPS corresponds to an inflow of water while an increase of QPS indicates an exit of water^26^,^31^.

Quantitative phase images of perfused neuronal cell cultures are obtained as previously described^31^,^32^. Briefly, holograms are acquired with a DHMT 1000 (Lyncée Tech SA, PSE-EPFL; Switzerland) using a laser diode source (λ = 682 nm) producing a coherent beam, which is divided by a beam splitter into a reference wave and an object wave. The object wave diffracted by the specimen is collected by a microscope objective and interferes with the reference beam, according to a Mach-Zehnder configuration, to produce the hologram recorded by the CCD camera. Frequency of hologram acquisition is 0.2 Hz. Reconstruction of the original image from the hologram is numerically achieved by a computer. According to Cuche *et al.*^33^ and Colomb *et al.*^34^, the reconstruction algorithm provides simultaneous amplitude and quantitative phase images of the cells (Koala software, Lyncée Tech SA, PSE-EPFL; Switzerland).

All cell cultures are perfused in a ACSF containing (in mM): NaCl 140, KCl 3, D-glucose 5, HEPES 10, CaCl_2_ 1.8, and MgCl_2_ 0.8 (pH 7.4; room temperature). For Mg^2+^ free ACSF, MgCl_2_ is substituted by a equimolar concentration of CaCl_2_ giving rise to a final concentration of 2.6 mM for Ca^2+^. All drugs are dissolved and applied around 20 min before the beginning of optical recordings. Note that when specified L-Lactate, pyruvate and D-lactate are added in the ACSF medium containing 5 mM of D-Glucose. The transient application of glutamate (100 μM; 2 min) are done after a minimum of 2 min of stable baseline recording of optical signal.

### Offline analysis

A minimum of 20 neurons per culture are analyzed for each experiment. The optical recordings are analysed by using MATLAB 7.6 (Mathworks Software, Natick, USA) and all curves are fitted by using ORIGIN 9.5 (Microcal Software, Northampton, USA).

### Cell viability assessment

For the cell viability assessment a modified configuration of the DHM T1000 microscope was used (Lyncée Tech SA, PSE-EPFL; Switzerland) that allows measurements of both QP and fluorescence signals in parallel^26^. Prior to experiments, coverslips were mounted on a closed chamber and cultures are perfused with the Mg^2+^ free ACSF containing (or not) L-Lactate at 10 mM. In this setup configuration, glutamate (1 mM; 5 min) was applied after a minimum of 2 min of stable baseline recording of QPS to induce excitotoxicity. Following QP-DHM imaging acquisition, cell viability is assessed with propidium iodide (PI) (Sigma-Aldrich, Buchs SG, Switzerland), a marker of cell death^35^,^36^. To this end, following a minimum period of 2.5 hours after glutamate application (time required for the occurrence of cellular death), PI (7.5 μM) was added in the perfusion medium during 15 minutes. At the end of the incubation, cells positive for PI staining, indicative of a loss of membrane integrity and cell death, were detected by epifluorescence microscopy. For the fluorescent system, excitation light is provided by a monochromator (Polychrome V, Till Photonics, Munich, Germany), delivering light ranging from 320 nm to 680 nm, with a power of typically 10 mW at 470 nm and a bandwidth of 15 nm. Employing a monochromator provides the possibility of switching rapidly the excitation wavelength without using any emission filter, thus avoiding any mechanical movement. The PI is excited at a single wavelength of λ = 490 nm and emits to 636 nm. The fluorescence light is then detected by an electron-multiplying CCD (iXon DU887, Andor Technology; Belfast, UK), cooled at −45 ^°^C and recording 16-bit images with exposure times of 1 second. The extraction of the two different signals is performed by employing two different dichroic mirrors, enabling first the separation of the line wavelength employed for digital holography, and second to enable the epifluorescence excitation^26^.

### Quantitative RNA analysis

Quantitative RNA analyses for pannexins and P2Y metabotropic purinergic receptor family members are performed using RNA-seq as part of a whole transcriptome study aimed to characterize basal gene expression levels in the neuronal cell culture model that is used in this study. Briefly, total RNA is isolated from DIV35 cultured neurons using Nucleospin RNA II kit (Macherey-Nagel; Oensingen, Switzerland) according to the manufacturer’s instructions. RNA quality is assessed on an Agilent 2100 Bioanalyzer (Agilent, Santa Clara, USA), and 1μg with RNA Integrity Number above 8 were used to construct libraries using the TruSeq Stranded mRNA Sample Kit (Illumina; San Diego, USA) following the protocol’s instructions. First, mRNA is enriched using oligo dT-attached magnetic beads, fragmented, and converted into cDNA. Next, these cDNA fragments go through an end repair process, 3′ ends are adenylated, universal bar-coded adapters are ligated, and cDNA fragments are amplified by 7–8 PCR cycles to yield the final libraries. These sequencing libraries are evaluated and quantified using an Agilent 2100 Bioanalyzer and qPCR. Paired-end read (2 × 100 bp) multiplex sequencing from pooled libraries is performed on an Illumina HiSeq 2000 machine at the KAUST Bioscience Core Labs. Data are processed according to the method described by Trapnell *et al.* (2012)^37^. Low-quality reads (QC < 30) are filtered and adapter sequences trimmed using SeqClean software. TopHat is then used to align the reads to the mus musculus genome. Finally, Cufflinks is used to assemble the aligned reads into transcripts and to estimate transcripts abundance in Fragment Per Kilobase of exon per Million fragments mapped (FPKM), which normalizes levels of transcripts for length and total number of reads.

### Statistical analysis

All data are presented as means ± SEM. Unpaired Student’s *t*-test or One-way ANOVA followed by Dunnett’s post hoc test (vs respective controls) have been used to determine statistical significance (*p* *<* 0.05).

## Results

### Glutamate triggers an irreversible decrease (ID) of phase shift specific for NMDA-dependent neuronal death

**Figure 1:**
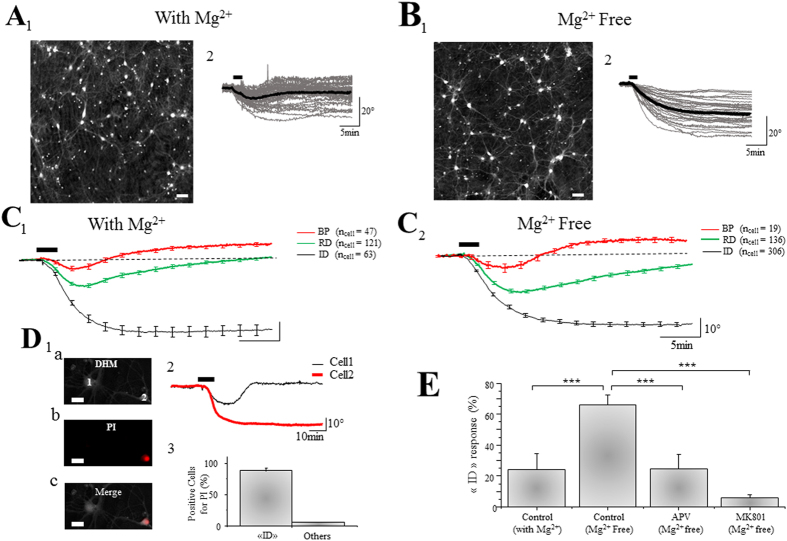
Excitotoxicity elicits a specific “ID” optical response in cultured neurons as revealed by QP-DHM. (**A**_**1**_,**B**_**1**_) Representative phase images of a neuronal culture perfused in the presence Mg^2+^(**A**_**1**_) or absence of Mg^2+^ (**B**_**1**_) (scale bar: 50 μm). (**A**_**2**_,**B**_**2**_) Individual (grey line) and averaged (black line) traces of QPS recorded from cultured neurons respectively in (**A**_**1**_,**B**_**1**_). In the presence of Mg^2+^, the averaged trace obtained after glutamate application (100 μM, 2 min; bar) is an “RD” response (**A**_**2**_) while, in the absence of Mg^2+^, the averaged trace is a “ID” response (**B**_**2**_). (**C_1_**,**C_2_**) Averaged traces of the 3 characteristic optical signals induced by glutamate application (100 μM, 2 min; bar) in the presence of Mg^2+^ (**C**_**1**_) or absence of Mg^2+^ (**C**_**2**_). There is no detectable difference in terms of shape and amplitude with phase responses obtained in Mg^2+^ condition. (**D**_**1**_) Visualization of 2 neurons in DHM (**a**) and in PI staining (**b**), the merged image showing that the Cell n°1 is negative for PI and cell n°2 positive for PI (**c**) (scale bar: 20 μm). (**D_2_**) Traces recorded in DHM from neurons in D_1_ indicates that the cell n°1, negative for PI, displays an “RD” response in contrast to cell n°2 positive for PI and displaying an “ID” response. (**D**_**3**_) The Bar chart shows percentage of cells positive for PI as a function of their phase response (n_cell_ = 66). (**E**) The Bar chart shows percentage of glutamate-induced “ID” responses obtained in different conditions: control with Mg^2+^ (n_cult_ = 8; n_cell_ = 231), control in Mg^2+^ free (n_cult_ = 16; n_cell_ = 461), with APV (50 μM) in Mg^2+^ free (n_cult_ = 8; n_cell_ = 223) and with MK801 (40 μM) in Mg^2+^ free.(n_cult_ = 8; n_cell_ = 228). Results are data are presented as means ± SEM (***p < 0.005, One-way Anova/post-test “Dunnett”).

As previously described, early signs of neuronal death elicited by glutamate-induced excitotoxicity can be accurately monitored by analysing quantative phase signals (QPS) using QP-DHM^26^,^27^. In our highly enriched neuronal cultures^29^ perfused with a ACSF containing Mg^2+^ (0.8 mM), transient application of glutamate (100 μM; 2 min) triggers 3 types of phase responses in cells, respectively defined as biphasic (BP), reversible decrease (RD) and irreversible decrease (ID) responses as revealed by DHM (Fig. 1A,C). The “BP” response is characterized by an initial decrease of the QPS (−6 + /−1.49°; n_cell_ = 47) followed by a recovery including a transient increase exceeding the initial basal level (10.8 + /−1.1°) (Fig. 1A,C). The “RD” response corresponds to a stronger initial decrease following by a complete recovery (−17.6 + /− 1.25°; n_cell_ = 121) (Fig. 1A,C). Lastly, the “ID” response corresponds to a pronounced and irreversible decrease of the QPS (−47.5 + /− 3.2°; n_cell_ = 63) (Fig. 1A,C). We have previously demonstrated that, in these experimental conditions, “BP” and “RD” responses corresponded to non-excitotoxic processes respectively associated to a mild and strong activation of NMDA receptors^26^. In these two cases, a delayed and full recovery of cell volume, hence of neuronal water and ionic homeostasis, was observed. In contrast, “ID” responses have been associated with excitotoxic activation of NMDA receptors and are characterized by an absence of cell volume recovery leading to cell death^26^,^27^. In the conditions used in this study (*i.e.* ACSF containing 0.8 mM Mg^2+^), the percentage of neurons displaying an “ID” response after a glutamate stimulation is moderate, 24 + /− 10% of cells, whereas 53 + /− 5% of neurons display an “RD” response and 23 + /− 5% for “BP” response (n_cult_ = 8; n_cell_ = 231).

We then tested the effect of the removal of Mg^2+^ from the extracellular solution on neuronal viability, a condition which promotes the excitotoxic activity of NMDA receptor by relieving NMDA Mg^2+^ block. In this specific condition, the percentage the “ID” responses induced by the same application protocol for glutamate (100 μM; 2 min) significantly increases to reach 66 + /− 6%, while “RD” and “BP” responses strongly decrease to respectively 29 + /− 5% and 5 + /− 2% (ncult = 16; ncell = 461) (Fig. 1B,C). In order to confirm the correlation between increased “ID” pattern and increased neuronal death, QPS recordings and propidium iodide (PI) staining, a marker of cell death^35^,^36^, was performed sequentially on the same neuronal preparations. It was found that 86% of neurons (57 out of 66 neurons) displaying an “ID” responses are also positive for PI (Fig. 1D), indicating unambiguously that “ID” response corresponds to an optical signature of the early phase of cell death process, as previously reported using PI or trypan blue staining^26^,^27^. Of note, the amplitude of these “ID” responses obtained in Mg^2+^-free solution (−42.4 + /− 1.28°; n_cell_  = 306) is similar to those obtained in Mg^2+^-containing medium (see above), strongly suggesting a shared mechanism of cell death between both conditions. Finally, APV (50 μM) and MK801 (40 μM), two specific antagonists of NMDA receptors, decrease the percentage of “ID” response respectively to 25 + /− 9% (n_cult_ = 8; n_cell_ = 223) and 6 + /− 2% (n_cult_ = 8; n_cell_ = 228) (Fig. 1E, Table 1) demonstrating that the cell death processes triggered by glutamate in Mg^2+^-free condition (as well as for Mg^2+^-containing condition, see above) are dependent upon the activation of NMDA receptors.

In the following experiments of this study, the “Mg^2+^-free condition” with an application of glutamate (100 μM, 2 min) was used as a standard experimental procedure to trigger “ID” responses.

### L-Lactate is neuroprotective against excitotoxicity: involvement of mitochondrial oxidative metabolism

**Figure 2:**
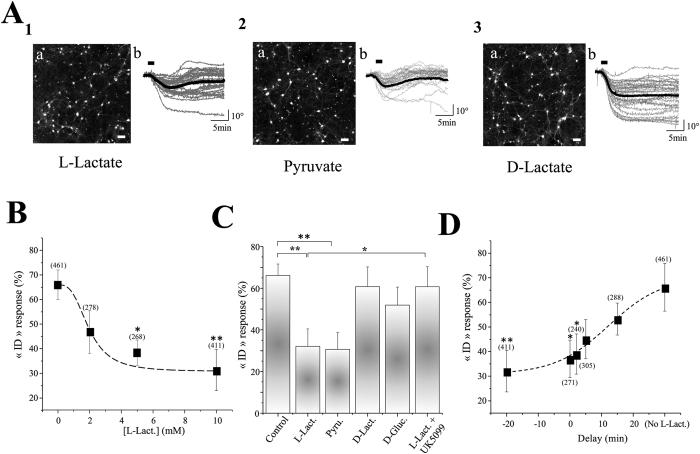
L-Lactate is neuroprotective through mitochondrial ATP formation. (**A**_**1**_) (**a**) Representative phase images of neuronal culture with L-Lactate (10 mM) in the Mg^2+^ free ACSF (scale bar: 50 μm). (**b**) Individual (grey line) and averaged (black line) traces of QPS recorded from cultured neurons. Note that the averaged trace obtained after glutamate application (100 μM, 2 min; bar) is an “RD” response. (**A**_**2**_**, A**_**3**_) Representative phase images (**a**), individual (grey line) and averaged (black line) traces of QPS (**b**) recorded from cultured neurons treated with Pyruvate (**A**_**2**_) or D-Lactate (**A**_**3**_). Similarly to L-Lactate, the averaged trace obtained after glutamate application is an “RD” response with Pyruvate. In contrast, the averaged trace of QPS is an “ID” response with D-Lactate. (**B**) Concentration-response curve representing the effect of L-Lactate at different (0 to 10 mM). Results are data are presented as means ± SEM (*p < 0.05, **p < 0.01, One-way Anova/post-test “Dunnett”). Numbers in brackets are the number of cells in each sampling. (**C**) The Bar chart shows percentage of glutamate-induced “ID” responses obtained in Mg^2+^ free ACSF containing 5 mM of D-glucose (control) (n_cult_ = 16; n_cell_ = 461), or supplied with L-Lactate (L-Lact., 10 mM) (n_cult_ = 15; n_cell_ = 411), Pyruvate (Pyru., 10 mM) (n_cult_ = 10; n_cell_ = 265). D-Lactate (D-Lact., 10 mM) (n_cult_ = 11; n_cell_ = 305), D-Glucose (D-Gluc., 5 mM) (n_cult_ = 10; n_cell_ = 286) or L-Lactate (10mM) + UK5099 (1μM) (n_cult_ = 9; n_cell_ = 248). Results are data are presented as means ± SEM (*p < 0.05; **p < 0.01, One-way Anova/post-test “Dunnett”). (**D)** Histogram representing the effect of L-Lactate at different time points (20 min before; 0 min, 2 min, 5 min and 15 min after) of glutamate application. Note that L-Lactate remains efficient to neuroprotect even when it is applied up to 2 min after glutamate application. Results are data are presented as means ± SEM (*p < 0.05; **p < 0.01, One-way Anova/post-test “Dunnett”). Numbers in brackets are the number of cells in each sampling.

In order to demonstrate its role as a neuroprotective agent, L-Lactate (10 mM) was added to the Mg^2+^-free ACSF. In these conditions, the percentage of neurons displaying a glutamate evoked “ID” response significantly decreases to reach 32 + /− 8% (n_cult_ = 15; n_cell_ = 411) (Fig. 2A–C; Table 1), while “RD” and “BP” responses increase respectively to 47 + /− 5% (p < 0.05) and 21 + /− 6% (p < 0.05). Moreover, while PI labelling following DHM recordings indicates around 59% PI positive cells (49 out of 84 neurons; n_cult_ = 5) in control condition, only 31% (17 out of 54 neurons; n_cult_ = 5) are positive for PI in presence of L-Lactate, clearly demonstrating that L-Lactate is neuroprotective against an excitotoxic insult. Finally, this effect was shown to be concentration-dependent starting to be significant at a concentration of 5 mM (Fig. 2B).

We observed that another monocarboxylate, Pyruvate (10 mM), mimicks the neuroprotective action of L-Lactate since only 31 + /− 8% (n_cult_ = 10; n_cell_ = 265) of neurons present a “ID” response following glutamate application (Fig. 2A,C; Table 1). In contrast, substitution of L-Lactate by D-Glucose at equicaloric concentration (5 mM) (n_cult_ = 10; n_cell_ = 286), results in a slightly, but not significant, decrease of “ID” response in comparison with control values (52 + /− 9% *vs* 66 + /− 6%) (Fig. 2C; Table 1); while perfusion of D-Lactate (10 mM), the non-metabolized enantiomer of L-Lactate, has no effect on the occurrence of “ID” response (61 + /− 10%; n_cult_ = 11; n_cell_ = 305) (Fig. 2A,C; Table 1). When considering that i) once imported into cells L-Lactate is converted to Pyruvate by Lactate Dehydrogenase, which is then transported into the mitochondria through Mitochondrial Pyruvate Carrier (MPC) and metabolically oxidized to produce ATP ii) several lines of evidence indicate extracellular L-Lactate as a preferred fuel for mitochondrial metabolism in neurons^38^,^39^, the above described results point to an important role of mitochondrial L-Lactate metabolism for neuroprotection.

In support of this view, UK5099 (1 μM), the more potent inhibitor of the MPC^40^, abolishes the neuroprotective action of L-Lactate with a percentage of “ID” response reaching up to 61 + /− 10% (n_cult_ = 9; n_cell_ = 248) (Fig. 2C; Table 1), further indicating that L-Lactate confers neuroprotection against excitotoxic insults by an increase in neuronal mitochondrial energy substrate metabolism. The MCP inhibitor UK5099 is also known to block plasma membrane monocarboxylate transporters (MCTs) but with K_i_ values that are at least 2–3 orders of magnitude higher than those observed for MCP inhibition (micromolar *vs* nanomolar range)^40^,^41^,^42^, which makes the concentration used in this study (1 μM) selective for MCP inhibition. In order to determine the time window during which L-Lactate remains effective to neuroprotect, L-Lactate is applied at different time points (0 min, 2 min, 5 min and 15 min) after glutamate application. L-Lactate maintains its neuroprotective property when applied up to 2 min after glutamate application (Fig. 2D).

### Extracellular-released ATP mediates L-Lactate-induced neuroprotection

**Figure 3:**
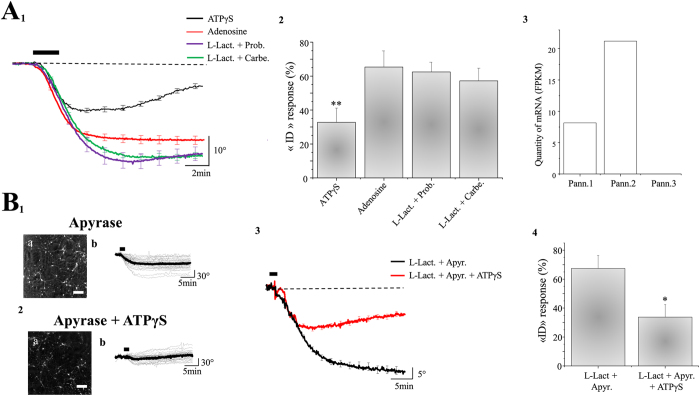
The neuroprotective effect of L-Lactate involves neuronal release of ATP through pannexins. (**A**_**1**_) With ATPγS (10 μM) in Mg^2+^ free ACSF, the averaged trace of QPS induced by glutamate (100 μM; 2 min) is an “RD” response (n_cult_ = 10; n_cell_ = 271) in contrast to adenosine (100 μM; n_cult_ = 10; n_cell_ = 261), L-Lactate (L-Lact., 10 mM) + Probenecid (Prob., 1 mM) (n_cult_ = 10; n_cell_ = 287) or L-Lactate + Carbenoxolone (Carbe., 100 μM) (n_cult_ = 10; n_cell_ = 285). (**A**_**2**_): Bar chart shows the percentage of glutamate-induced “ID” responses obtained in different conditions described in A_1_. Results are presented as means ± SEM (**p < 0.01, One-way Anova/post-test “Dunnett”). (**A**_**3**_): Histogram represents the measurement of mRNA for the 3 isoforms of Pannexin (Pann.1, Pann.2 and Pann.3) expressed in neuronal culture. Note the absence of mRNA of Pann.3. (**B**) Two representative phase images of neuronal cultures (scale bar: 50 μm) perfused with L-Lactate (10 mM) in the presence of 30U/ml of apyrase (**B**_**1a**_) or apyrase (30 U/ml) + ATPγS at 10 μM (**B**_**2a**_), each image being accompanied by the individual (grey line) and averaged (black line) traces of QPS recorded from cultured neurons (**B**_**1b**_,**B**_**2b**_). (**B**_**3**_) The averaged trace induced by glutamate application (100 μM, 2 min; bar) in the presence of L-Lactate and apyrase (Apyr.) alone (n_cult_ = 9; n_cell_ = 210) is an “ID” response while addition of ATPγS promotes an “RD” response (n_cult_ = 9; n_cell_ = 219). (**B**_**4**_) Bar chart summarizing the effects of apyrase (with or without APTγS) on the occurrence of “ID” responses . Results are presented as means ± SEM (*p < 0.05; Unpaired *t*-test).

The previous set of data unambiguously showed that the protective effect of L-Lactate against excitotoxic injury is associated with mitochondrial oxidative metabolism pathway, suggesting that the neuroprotective mechanism is associated with the production of ATP. This observation raises the question of the mechanisms by which ATP can protect neurons against a glutamate excitotoxicity? Two main hypotheses are considered : i) ATP is directly used as an energy store to sustain cell functions (essentially to maintain ionic homeostasis by ATP-dependent pumps or others proteins) and/or ii) ATP acts as a signaling molecule through the activation of purinergic receptors. As a first approach aiming to delineate which of these hypotheses is valid, the effect of ATPγS, a non-hydrolyzable form of ATP, was tested. While ATPγS is a stable agonist of different purinergic receptors (see below) it is also a useful tool to exclude the involvement of energy-dependent ATP processes. Indeed, ATPγS cannot be used as phosphate-bound energy donor for processes requiring ATP cleavage to ADP such as ATP-dependent ion pumps. In the presence of ATPγS (10 μM) in Mg^2+^ free ACSF, the averaged phase response displayed by neurons (n_cell_ = 271) is a “RD”-type response (Fig. 3A), indicating that a minority of neurons underwent excitotoxic damage after application of glutamate. For instance, only 33 + /− 9% of neurons of the 10 studied cultures display an “ID” response (Fig. 3A), in contrast to the 66 + /− 6% observed in control conditions (Fig. 3A; Table 1). These experiments, stress the fact that ATP is involved in this neuroprotective mechanism as a signaling molecule acting on purinergic receptors rather than as energy donor.

Once released into the extracellular space, ATP is degraded by ectopeptidases to the purine nucleoside adenosine. Both ATP and adenosine have been previously shown (in particular adenosine) to exert neuroprotective effects through activation of selective subtypes of purinergic receptors, notably P1 and P2 receptors for adenosine and ATP respectively^43^,^44^.

Since ATPγS, cannot be cleaved to ADP (and therefore in AMP and adenosine), this L-Lactate-like effect suggests that P2 receptors are a major player in the neuroprotection induced by L-Lactate. In support of this, in the presence in the extracellular medium of PPADS (30 μM), a broad-spectrum blocker of P2 receptors, neuroprotection induced by L-Lactate against glutamate excitotoxicity is abolished (“ID” response 58 + /− 9%; n_cult_ = 10; n_cell_ = 277) (Table 1). Furthermore, incubation of adenosine (100μM), does not reproduce the neuroprotective effects of L-Lactate or ATPγS (65 + /− 9% of “ID” response; n_cult_ = 10; n_cell_ = 261) ruling out the involvement of P1 adenosine receptors in this process (Fig. 3A). Altogether, these results strongly suggest that ATP (and not adenosine) acts as a signaling molecule through an activation of P2 receptors for protection against a glutamate excitotoxicity.

To obtain further insights on the ATP-dependent activation of P2 receptors, neuronal cultures were pre-treated with both, L-Lactate (10 mM) and apyrase (30 U/ml), a soluble ecto-nucleotidase degrading the extracellular ATP. In this condition, the protective effect of L-Lactate following application of glutamate is abolished, with a percentage of “ID” response of 67 + /− 9% (n_cult_ = 9; n_cell_ = 210) (Fig. 3B) not significantly different from control values (p > 0.05). Finally, co-application of ATPγS (10 μM) together with L-Lactate and apyrase is able to re-induce neuroprotection with only 34 + /− 9% of “ID” responses induced by glutamate exposure (n_cult_ = 9; n_cell_ = 219) (Fig. 3B; Table 1). All these results indicate that ATP (and not its metabolites) produced by L-Lactate mitochondrial metabolism and released in the extracellular space induces neuroprotection by acting on P2 receptors.

A possible mechanism responsible for mediating neuronal extracellular ATP release involves pannexins, which are proteins expressed in neurons^45^ and known to play a role as ATP channels^46^. Quantitative expression of mRNA levels of pannexins isoforms in our cultures system were first determined by RNA-seq. Data shown in Fig. 3A indicate that mRNA for pannexin 1 and 2 are abundantly expressed in these cultured neurons. Both pannexins blockers probenicid (1mM) and carbenoxolone (10μM) inhibit the neuroprotective effect of L-Lactate with a percentage of “ID” response (induced by glutamate) of 62 + /− 6% (n_cult_ = 10; n_cell_ = 287) and 57 + /− 7% (n_cult_ = 10; n_cell_ = 285) respectively (Fig. 3A; Table 1), thus indicating that pannexins are the channels through which ATP produced by the L-Lactate/Pyruvate pathway are released.

### The neuroprotective effect of L-Lactate involves a P2Y/PI3K cascade

**Figure 4:**
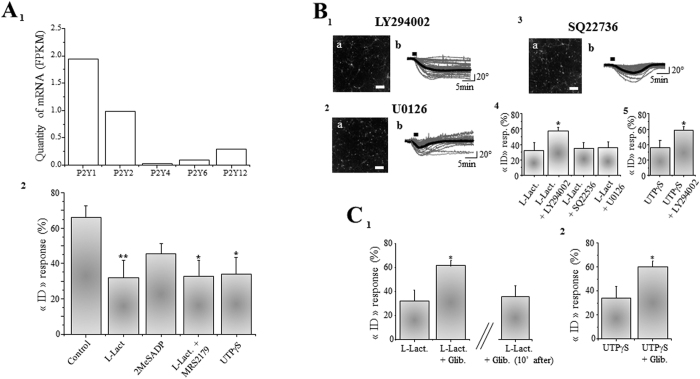
A P2Y2/PI3K/K_ATP_ cascade mediates the neuroprotective effect of L-Lactate. (A_**1**_) Histogram representing the determination of mRNA for different isoforms of P2Y receptors. P2Y1 and P2Y2 are the two major isoforms expressed in neuronal cultures. (**A**_**2**_) The Bar chart shows percentage of glutamate-induced “ID” responses obtained in control condition (n_cult_ = 16; n_cell_ = 461), with L-Lactate (L-Lact., 10mM) (n_cult_ = 15; n_cell_ = 411), 2MeSADP (10μM) (n_cult_ = 10; n_cell_ = 280), L-Lactate + MRS2179 (30 μM) (n_cult_ = 10; n_cell_ = 273) and UTPγS (10 μM) (n_cult_ = 10; n_cell_ = 276). Note the significant effect of UTPγS, the agonist of P2Y2 receptor. (**B**) Three representative phase images of neuronal cultures (scale bar: 50 μm) perfused with L-Lactate (10mM) in the presence of LY294002 (10 μM) (**B**_**1a**_), U0126 (10μM) (**B**_**2a**_), or SQ22736 (100μM) (**B**_**3a**_), each image being accompanied by the individual (grey line) and averaged (black line) traces of QPS recorded from cultured neurons and the histograms indicating the number of cells classified as a function of their optical responses (**B**_**1b**_,**B**_**2b**_,**B**_**3b**_). (**B**_**4**_) Bar chart summarizing the effects of LY294002 (n_cult_ = 10; n_cell_ = 283), SQ22536 (n_cult_ = 10; n_cell_ = 248) and U0126 (n_cult_ = 10; n_cell_ = 273) on the occurrence of “ID” responses in the presence of L-Lactate in Mg^2+^-free ACSF. Only LY294002 significantly blocked the effect of L-Lactate. (**B**_**5**_) Bar chart comparing the effect of UTPγS (10 μM) on the percentage of “ID” response in the presence (n_cult_ = 11; n_cell_ = 312) or absence (n_cult_ = 10; n_cell_ = 276) of LY294002. (**C**_**1**_) Bar chart shows the significant blockage of glibenclamide (Glib., 10 μM) on the neuroprotective effects of L-Lactate (n_cult_ = 10; n_cell_ = 283). This blocking effect disappears when glibenclamide is applied 10 min after the application of glutamate (ncult = 10; ncell = 280). (**C**_**2**_) Bar chart reports the significant blockage of glibenclamide (10 μM) on the neuroprotective effects of UTPγS (10μM) (n_cult_ = 10; n_cell_ = 296). For **A**_**2**_, **B**_**4**_, and **C**_**1**_, results are presented as means ± SEM (*p < 0.05; **p < 0.01, One-way Anova/post-test “Dunnett”). For (**B**_**5**_,**C**_**2**_), results are presented as means ± SEM (*p < 0.05; Unpaired *t*-test).

The P2 receptor family comprises two subtypes, the ionotropic P2X, a family of ATP-gated cation channels, and the metabotropic P2Y, a family of G-protein-coupled receptors. (G-protein-coupled receptors)^43^,^47^. However, the fact that P2X receptors are non-selective cation channels with high Ca^2+^ permeability does not argue in favour of an active role of these receptors in neuroprotection (*i.e.* activation of P2X will produce a calcium inflow additional to that of NMDA activation) which is consistent with observations made in several studies demonstrating that P2X receptors activation is deleterious for neuronal survival^48^,^49^. These observations lead us to consider the metabotropic P2Y receptor family as a likely mediator of L-Lactate protective effects. Quantitative analysis of mRNA levels for P2Y family members indicates that mRNA for P2Y1 and P2Y2 receptors are abundantly expressed in our primary cultures of cortical neurons preparations (Fig. 4A).

We observe that UTPγS (10μM), a specific agonist for P2Y2 receptor, significantly protects neurons against glutamate excitotoxicity with a percentage of “ID” responses close to 34 + /− 9% (n_cult_ = 10; n_cell_ = 276) (Fig. 4A and Table 1), suggesting that the neuroprotective activity of L-Lactate is associated with ATP-mediated activation of P2Y2 receptors.

Consistent with this view, 2MeSADP (10 μM), a specific agonist of P2Y1 receptor, has no neuroprotective effect, since the percentage of neurons displaying an “ID” response after the application of glutamate is 46 + /− 6% (n_cult_ = 10; n_cell_ = 280) (Fig. 4A; Table 1), a value lower that glutamate alone but not significantly different from control values (Fig. 4A). Consistent with the lack of involvement of P2Y1 receptors, the specific antagonist of P2Y1 receptor, MRS 2179 (30 μM) has no inhibitory action on the neuroprotective effect of L-Lactate (33 + /− 9%; n_cult_ = 10; n_cell_ = 273) (Fig. 4A and Table 1).

P2Y2 receptors are G protein-coupled receptors activating numerous intracellular pathways^47^,^50^, including the phosphatidylinositol 3' –kinase (PI3K) cascade associated with the activation of different neuronal survival pathways^51^. Interestingly, in the presence of LY294002 (10 μM), a specific blocker of PI3K, the protective effects of L-Lactate are prevented with a percentage of “ID” responses observed in 57 + /− 5% of neurons perfused with LY294002 and L-Lactate (n_cult_ = 10; n_cell_ = 283) (Fig. 4B; Table 1), a value significantly higher than for neurons treated with L-Lactate alone (Fig. 4B). This result strongly suggests that the neuroprotective effect of L-Lactate is linked to the activation of the PI3K cascade. In contrast, in the presence of SQ22536 (100 μM), a blocker of the Adenylate Cyclase pathway or U0126 (10 μM), a blocker of the MAP Kinase pathway, the neuroprotective effects of L-Lactate persist (Fig. 4B and Table 1) indicating the specific involvement of PI3K in the L-Lactate-induced neuroprotection.

Finally, when neuronal cultures (n_cult_ = 11; n_cell_ = 312) are treated with LY294002 (10μM) and UTPγS (10 μM) in combination, 56 + /− 5% of cells display an “ID” responses after application of glutamate (Fig. 4B; Table 1), a percentage of “ID” response significantly higher than with UTPγS alone (Fig. 4B), further confirming the involvement of PI3K in the L-Lactate-induced P2Y2 receptors-mediated neuronal survival pathway.

### PI3K-dependent neuronal survival pathway is associated to an opening of an ATP-sensitive potassium channel (K_ATP_)

Neuronal survival elicited by the activation of the PI3K signaling pathway is mostly associated with long-term inhibitory effects on the expression of pro-apoptotic factors as well as with promoting action on the expression of anti-apoptotic factors^52^. As our experimental settings rely on the detection of early signs of excitotoxic cell death *i.e.* neuronal cell swelling occurring within minutes following glutamate application, PI3K long-term effects on the expression of apoptotic factors do not appear to be mediators of such L-Lactate-mediated short-term effects. Furthermore, this notion of immediacy is reinforced by the fact that, as shown in Fig. 2D, the neuroprotective effect of L-Lactate is observed only when it applied with a maximum delay of 2 min after the onset of glutamate exposure.

In the context of rapid PI3K-dependent mediators of neuroprotection, K_ATP_ channels have emerged as potential candidates to account for the effects of L-Lactate. K_ATP_ channels are indeed involved in neuroprotection processes^53^ and are regulated (*i.e.* activated) by the PI3K pathway^54^ via reduction of their sensitivity to ATP^55^. This in turn leads to a decrease in neuronal excitability, which in the context of glutamate-evoked excitotoxicity may be beneficial. In order to determine the importance of K_ATP_ channels in the effects of L-Lactate, glibenclamide, an inhibitor of K_ATP_ channel was used. Perfusion of neuronal cultures in the presence of L-Lactate (10 mM) and glibenclamide (10 μM), results in a significant decrease of the neuroprotective effects of L-Lactate with a percentage of “ID” responses induced by glutamate of 62 + /− 5% (n_cult_ = 10; n_cell_ = 283) (Fig. 4C; Table 1). Similarly, the neuroprotective effect of P2Y2 receptor agonist UTPγS (10 μM) is lost in the presence of glibenclamide (10 μM) since the percentage of “ID” responses reaches up 60 + /− 4% (n_cult_ = 10; n_cell_ = 296) (Fig. 4C). Of note, glibenclamide is no more effective in blocking L-Lactate effect when added 10min after glutamate application (ncult = 10; ncell = 280) (Fig. 4C). These results highlight the importance of the P2Y2/PI3K pathway and the activation of K_ATP_ channel in the L-Lactate-induced neuroprotective actions. Altogether these results demonstrate that the neuroprotective effect of L-Lactate against the excitotoxic action of glutamate is linked to the final activation of the K_ATP_ channel through the mobilization of the P2Y2/PI3K pathway.

## Discussion

**Figure 5:**
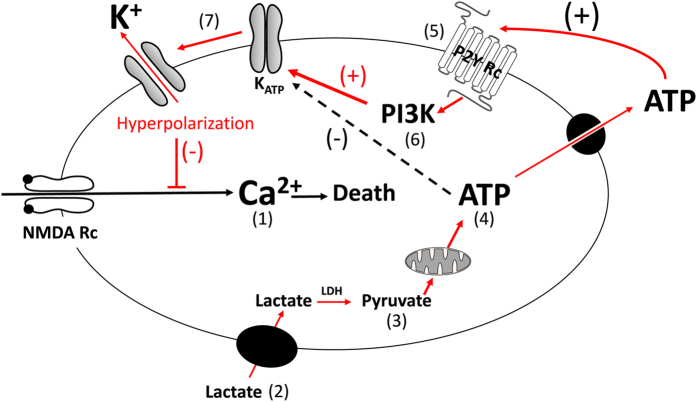
Schematic representation of the mechanisms involved in the neuroprotective effect of L-Lactate against excitotoxicity. Over stimulation of NMDA receptors by glutamate triggers a strong inflow of Ca^2+^; this overload of Ca^2+^ leading to excitotoxic cell death processes (1). L-Lactate is transported into the cell (2) and converted to Pyruvate by Lactate Dehydrogenase (LDH) (3). Pyruvate is transported into mitochondria through the Mitochondrial Pyruvate Carrier (MPC) to produce ATP (4). ATP is then released through pannexins and acts on the metabotropic purinergic receptor P2Y, likely P2Y2, in a autocrine/paracine manner (5). Stimulation of purinergic receptors activates the PI3K pathway (6) which, in turn, elicits the opening of K_ATP_ channels, hence leading to hyperpolarization of neurons (7), the consequence being a decrease in neuronal excitability leading to neuroprotection.

Initial phases of cellular death triggered by an excessive glutamate stimulation are characterized by a massive ionic and water inflows^56^. Taking advantage of the QP-DHM technique to monitor transmembrane water fluxes associated with early stages of neuronal death processes^26^,^27^, we demonstrate that L-Lactate acts as a signaling molecule conferring neuroprotection against excitotoxic insults through well-coordinated mechanisms based on an increase neuronal energy substrates availability, a release of ATP and an intracellular signaling PI3 kinase pathway triggered by purinergic receptors, likely P2Y2, followed by the activation of the K_ATP_ channels as summarized in Fig. 5.

Until now, the few *in vitro* studies exploring the neuroprotective properties of L-Lactate have suggested a mechanism of action involving the maintenance of the cellular energy charge^18^. Indeed, excitotoxicity is classically associated with inhibition of oxidative phosphorylation resulting in a loss of ATP to fuel ion pumps to re-establish the ionic homeostasis^9^,^10^. In agreement with that, the involvement of the L-Lactate/Pyruvate pathway and the mitochondrial activity was also observed in this study. Since L-Lactate is converted to Pyruvate by Lactate Dehydrogenase, it would seem logical that both, L-Lactate and Pyruvate are equivalent to produce ATP through the oxidative pathway (Fig. 5). In contrast, glucose does not evoke a significant neuroprotective effect consistent with the view that L-Lactate is a preferred fuel for mitochondrial metabolism in neurons^38^,^39^ and also in view of the fact that glutamate inhibits glucose transport in neurons^57^. Such view is supported by *in vivo* studies demonstrating the involvement of a L-Lactate/Pyruvate pathway for neuroprotection^13^,^58^,^59^. In contrast to these observations and to our present results, a recent study evidenced a neuroprotective role of D-lactate and L-lactate through a common mechanism involving energy production and activation of HCA1, a lactate receptor, suggesting that different D/L-lactate-induced neuroprotective pathways may operate *in vivo*^60^.

Another important data reported in the present study indicate the existence of an additional mechanism independent of an energetic role of L-Lactate linked to the formation of ATP since ATPγS, a non-hydrolysable ATP, mimicked effect of L-Lactate (and of Pyruvate). Indeed data indicate that ATP produced by the L-Lactate/Pyruvate neuroenergetic pathway acts as signaling molecule following its release through the ATP channels pannexins, a mode of release in agreement with the biophysical properties of the pannexins known to be a mechanosensitive conduits for ATP sensitive to swelling^46^,^61^. Interestingly, ATP released by neurons acts in autocrine/paracrine manner triggering an apyrase-sensitive purinergic signaling (Fig. 5). Such ATP-signaling cascade has been observed occurring not only in neurons^62^ but also in astrocytes^63^, these glial cells expressing both pannexins and purinergic receptors^64^,^65^. This could suggest that the very few contaminating astrocytes in our neuronal cultures model may also participate in the activation of such a signaling cascade. Nevertheless, while we cannot fully exclude a participation of astrocytes the biological effects of L-Lactate described here, the high enrichment of neurons in our culture model, 93%^29^, and therefore the low level of contaminating astrocytes, less than 7% of glial cells, makes it unlikely.

Concerning the nature of ATP-activated receptors, it is known that both, P1 adenosine and P2 purinergic receptors are involved in neuroprotection^66^. Thus, a neuroprotective role of adenosine through the activation of specific P1 receptors (i.e. A1) has been demonstrated, which was shown to be dependent on a decrease in extracellular glutamate release associated to an inhibition of excitatory synaptic transmission^67^,^68^,^69^. However, the neuroprotective effect of ATPγS along with the use of specific agonists and antagonists clearly indicates that the receptors involved in the effects described here are not adenosine receptors. The blockade of L-Lactate neuroprotection by apyrase confirms the pivotal role played by ATP originating from the L-Lactate/Pyruvate pathway through activation of P2 purinergic receptors, mostly the metabotropic P2Y family since ionotropic P2X receptors are non-selective cation channels with high Ca^2+^ permeability, an argument which is not in favour of an active role of these receptors in neuroprotection. It has been shown that ATP can exert neuroprotective actions through activation of neuronal purinergic P2Y2 receptors in a serum starvation-induced model^51^. P2Y subunits are known to potentially form homomeric or heteromeric assemblies with distinct types of GPCRs through direct association, providing complex non-canonical pharmacological responses to agonists/antagonists and downstream signaling pathways^70^, a fact that may preclude the identification of the role played by a given P2 receptor subtype in a physio(patho)logical process. Nevertheless, results obtained in this study and in particular the observation that UTPγS, a specific activator of P2Y2 receptor mimics the protective effects of L-Lactate points to a major role played by this receptor subtype in L-Lactate-mediated neuroprotection against excitotoxicity. An interesting point concerns a putative dual role played by ATP in neurotoxic and neuroprotective processes. Indeed, excessive release of ATP is known to promote neuronal death, in particular through the activation of a P2X7-pannexins complex^71^. Purinergic receptors display a very broad range of ATP sensitivities, ranging from nanomolar in the case of P2Y receptors up to hundred micromolar for P2X receptors^72^, activation of these receptors may therefore be associated with neuroprotective or neurotoxic effects respectively. This implies that depending on the ATP concentration reached in the extracellular medium different P2 receptors subtypes may be activated, hence either neuroprotective or neurotoxic cascades.

The P2Y2/PI3K pathway has been described as an important survival pathway through an inhibition of apoptotic processes^51^. Here we show, that the P2Y2/PI3K pathway can also promote neuronal survival through a rapid and non-genomic action involving the activation of the K_ATP_ channel (Fig. 5). Such interactions between PI3K pathway and K_ATP_ channels have been described in hypothalamic proopiomelanocortin neurons participating in the inhibition of food intake^55^. In particular, it was demonstrated that PI3K pathway enhances PIP3 signaling, resulting in the activation of K_ATP_ channels via reduction of the channel sensitivity to ATP. This type of intracellular mechanism resulting in the activation of K_ATP_ channels and prevention of depolarization, may also be related to neuroprotection. Indeed, while ATP production is necessary to maintain the activity of ATP-dependent ion pumps to restore the ionic balance disrupted by an overstimulation of glutamate receptors, this increase in ATP levels can also lead to a closure of K_ATP_ channels, with final result being a neuronal depolarization and, as a consequence, the maintenance of the neuronal death processes (Fig. 5). The PI3K-dependent process that we have revealed would therefore play a protective role by activating K_ATP_ channels despite an increase in intracellular ATP levels produced by L-Lactate/Pyruvate mitochondrial oxidation. The involvement of PI3K pathway appears to be critical for neuronal survival because ATPγS (or UTPγS) alone are sufficient to provide neuroprotection.

Other studies have shown that L-Lactate (and Pyruvate) exert their protecting effect by decreasing the delayed accumulation of extracellular glutamate known to be associated with excitatory insults induced by long-term exposure to NMDA^18^. Our results suggest that the activation of K_ATP_ channels by the P2Y2/PI3K pathway can be the underlying mechanism responsible for the decrease in the delayed glutamate accumulation described above by reducing the neuronal network excitability, hence decreasing global glutamate release. Interestingly, we have previously demonstrated that the shape and amplitude of optical responses triggered by glutamate application reflected the excitability of the neuronal network, particularly the level of NMDA activity^26^. In this general context, it would be of interest to determine whether differences in terms of levels of expression and activation of the key elements involved in the neuroprotective cascade induced by L-Lactate as described in this study could explain (or be involved in) the differential physiological response of neurons to L-Lactate (*i.e.* “RD” *vs* “ID” neurons).

In addition to its role in energy metabolism, there is growing evidence that L-Lactate can act as signaling molecule^12^,^73^,^74^ in particular during physiological processes like Long-Term Memory formation^75^. There is an apparent paradox in the effects of L-Lactate , as two contrasting effects appear to exist: a stimulation of plasticity and learning mediated by potentiation of the NMDA receptor activity^73^ which coexists with a neuroprotective effect of L-Lactate against excessive NMDA receptor activity (excitotoxicity). As shown here, it is actually conceivable that the coexistence of these two effects represents a coordinated and well balanced set of mechanisms that allows to potentiate NMDA receptor activity while mitigating its potential pernicious effects. The temporal deployment of these two effects would be consistent with this view, as the neuroprotective effect is rapidly expressed, within 2 minutes (this study), while the effect on plasticity is delayed in time between 30 minutes and 4 hours depending on the genes induced^73^.

Our study provides new evidences that L-Lactate can also act as a signaling molecule in pathological contexts such as excitotoxic processes. Considering that astrocytes are the main producers of L-Lactate in brain, our observations point to astrocytes as pivotal cellular elements for neuronal protection against excitotoxicity. For instance, glutamate released during neuronal activity stimulates glucose uptake and L-Lactate production and release from astrocytes^11^. Boosting this process, also known as aerobic glycolysis or Warburg effect^12^ indeed results in neuroprotection^76^. Therefore, during an excitotoxic situation, the pathological release of glutamate from neurons would strongly activate L-Lactate production and the release from astrocytes which, in turn, would provide neuroprotection by opening K_ATP_ channels, through the P2Y2/PI3K pathway.

In summary, the present results indicate that L-Lactate can be an attractive candidate as a neuroprotective compound, providing the opportunity to develop neuroprotective strategies aimed at increasing the production of L-Lactate by astrocytes.

## Additional Information

**How to cite this article**: Jourdain, P. *et al.* L-Lactate protects neurons against excitotoxicity: implication of an ATP-mediated signaling cascade. *Sci. Rep.* **6**, 21250; doi: 10.1038/srep21250 (2016).

## Figures and Tables

**Table 1:** Summary of the rate of “ID” responses induced by glutamate in different conditions.

Condition	n_cult_	n_cell_	≪ ID ≫ response (%)
Control	16	461	66.17 + /− 5.50
APV (50 μM)	8	223	24.65 + /− 9.43***
MK801 (40 μM)	8	228	5.85 + /− 2.07***
L-Lactate (10 mM)	15	411	32.08 + /− 8.44**
Pyruvate (10 mM)	10	265	30.64 + /− 8.14**
D-Lactate (10 mM)	11	305	60.76 + /− 9.52 *ns*
D-Glucose (5 mM)	10	286	51.90 + /− 9.16 *ns*
L-Lactate (10 mM) + UK5099 (1 μM)	9	248	60.71 + /− 9.70 *ns*
ATPγS (10 μM)	10	271	32.68 + /− 8.51**
Adenosine (100 μM)	10	261	65.33 + /− 8.97 *ns*
L-Lactate (10 mM) + Apyrase (30 U/ml)	9	210	67.40 + /− 8.89 *ns*
L-Lactate (10 mM) + Apyrase (30 U/ml) + ATPγS (10 μM)	9	219	33.74 + /− 8.71**
L-Lactate (10 mM) + PPADS (30 μM)	10	277	57.94 + /− 9.12 *ns*
2MeSADP (10 μM)	10	280	45.62 + /− 5.86 *ns*
L-Lactate (10mM) + MRS 2179 (30 μM)	10	273	33.08 + /− 8.70*
UTPγS (10 μM)	10	276	34.19 + /− 9.46*
L-Lactate (10 mM) + Probenecid (1 mM)	10	287	62.46 + /− 5.68 *ns*
L-Lactate (10 mM) + Carbenoxolone (10 μM)	10	285	57.15 + /− 7.45 *ns*
L-Lactate (10 mM) + SQ22532 (100 μM)	10	248	34.63 + /− 7.60***
L-Lactate (10 mM) + U0126 (10 μM)	10	273	35.69 + /− 7.91***
L-Lactate (10 mM) + LY294002 (10 μM)	10	283	57.44 + /− 5.17 *ns*
UTPγS (10 μM) + LY294002 (10 μM)	11	312	56.06 + /− 5.03 *ns*
L-Lactate (10 mM) + Glibenclamide (10 μM)	10	283	62.39 + /− 5.47 *ns*
UTPγS (10 μM) + Glibenclamide (10 μM)	10	296	60.30 + /− 4.41 *ns*

Values are means ± SEM. Statistics data correspond to a Dunnett’s post hoc test following a one-way ANOVA (*ns*: p > 0.05; *p < 0.05; **p < 0.01; ***p < 0.005).

All presented results are obtained in Mg^2+^ free ACSF.

## References

[b1] Olney J. W., Price M. T., Samson L. & Labruyere J. The role of specific ions in glutamate neurotoxicity. Neurosci Lett. 65, 65–71 (1986). 2871531 10.1016/0304-3940(86)90121-7

[b2] Sattler R. & Tymianski M. Molecular mechanisms of glutamate receptor-mediated excitotoxic neuronal cell death. Mol Neurobiol. 24, 107–129, doi: 10.1385/MN:24:1-3:107 (2001). 11831548

[b3] Park E., Velumian A. A. & Fehlings M. G. The role of excitotoxicity in secondary mechanisms of spinal cord injury: a review with an emphasis on the implications for white matter degeneration. J Neurotrauma. 21, 754–774, doi: 10.1089/0897715041269641 (2004). 15253803

[b4] Lai T. W., Zhang S. & Wang Y. T. Excitotoxicity and stroke: identifying novel targets for neuroprotection. Prog Neurobiol. 115, 157–188, doi: 10.1016/j.pneurobio.2013.11.006 (2014). 24361499

[b5] Hynd M. R., Scott H. L. & Dodd P. R. Glutamate-mediated excitotoxicity and neurodegeneration in Alzheimer's disease. Neurochem Int. 45, 583–595, doi: 10.1016/j.neuint.2004.03.007 (2004). 15234100

[b6] Van Den Bosch L., Van Damme P., Bogaert E. & Robberecht W. The role of excitotoxicity in the pathogenesis of amyotrophic lateral sclerosis. Biochim Biophys Acta. 1762, 1068–1082, doi: 10.1016/j.bbadis.2006.05.002 (2006). 16806844

[b7] Doble A. The role of excitotoxicity in neurodegenerative disease: implications for therapy. Pharmacol Ther. 81, 163–221 (1999). 10334661 10.1016/s0163-7258(98)00042-4

[b8] Muir K. W. Glutamate-based therapeutic approaches: clinical trials with NMDA antagonists. Curr Opin Pharmacol. 6, 53–60, doi: 10.1016/j.coph.2005.12.002 (2006). 16359918

[b9] Rodriguez-Rodriguez P., Almeida A. & Bolanos J. P. Brain energy metabolism in glutamate-receptor activation and excitotoxicity: role for APC/C-Cdh1 in the balance glycolysis/pentose phosphate pathway. Neurochem Int. 62, 750–756, doi: 10.1016/j.neuint.2013.02.005 (2013). 23416042

[b10] Connolly N. M. & Prehn J. H. The metabolic response to excitotoxicity - lessons from single-cell imaging. J Bioenerg Biomembr. 47, 75–88, doi: 10.1007/s10863-014-9578-4 (2015). 25262286

[b11] Pellerin L. & Magistretti P. J. Glutamate uptake into astrocytes stimulates aerobic glycolysis: a mechanism coupling neuronal activity to glucose utilization. Proc Natl Acad Sci USA. 91, 10625–10629 (1994). 7938003 10.1073/pnas.91.22.10625 PMC45074

[b12] Magistretti P. J. & Allaman I. A Cellular Perspective on Brain Energy Metabolism and Functional Imaging. Neuron 86, 883–901, doi: 10.1016/j.neuron.2015.03.035 (2015). 25996133

[b13] Ros J., Pecinska N., Alessandri B., Landolt H. & Fillenz M. Lactate reduces glutamate-induced neurotoxicity in rat cortex. J Neurosci Res. 66, 790–794 (2001). 11746403 10.1002/jnr.10043

[b14] Izumi Y. & Zorumski C. F. Neuroprotective effects of pyruvate following NMDA-mediated excitotoxic insults in hippocampal slices. Neurosci Lett. 478, 131–135, doi: 10.1016/j.neulet.2010.04.078 (2010). 20452397 PMC2913700

[b15] Berthet C. *et al.* Neuroprotective role of lactate after cerebral ischemia. J Cereb Blood Flow Metab. 29, 1780–1789, doi: 10.1038/jcbfm.2009.97 (2009). 19675565

[b16] Horn T. & Klein J. Neuroprotective effects of lactate in brain ischemia: dependence on anesthetic drugs. Neurochem Int. 62, 251–257, doi: 10.1016/j.neuint.2012.12.017 (2013). 23298645

[b17] Berthet C., Castillo X., Magistretti P. J. & Hirt L. New evidence of neuroprotection by lactate after transient focal cerebral ischaemia: extended benefit after intracerebroventricular injection and efficacy of intravenous administration. Cerebrovasc Dis. 34, 329–335, doi: 10.1159/000343657 (2012). 23154656

[b18] Maus M., Marin P., Israel M., Glowinski J. & Premont J. Pyruvate and lactate protect striatal neurons against N-methyl-D-aspartate-induced neurotoxicity. Eur J Neurosci. 11, 3215–3224 (1999). 10510185 10.1046/j.1460-9568.1999.00745.x

[b19] Bowser D. N. & Khakh B. S. ATP excites interneurons and astrocytes to increase synaptic inhibition in neuronal networks. J Neurosci. 24, 8606–8620, doi: 10.1523/JNEUROSCI.2660-04.2004 (2004). 15456834 PMC6729897

[b20] Bozzo L., Puyal J. & Chatton J. Y. Lactate modulates the activity of primary cortical neurons through a receptor-mediated pathway. PloS one 8, e71721, doi: 10.1371/journal.pone.0071721 (2013). 23951229 PMC3741165

[b21] Tang F. *et al.* Lactate-mediated glia-neuronal signalling in the mammalian brain. Nat Commun. 5, 3284, doi: 10.1038/ncomms4284 (2014). 24518663 PMC3926012

[b22] Morland C. *et al.* The lactate receptor, G-protein-coupled receptor 81/hydroxycarboxylic acid receptor 1: Expression and action in brain. J Neurosci Res. 93, 1045–1055, doi: 10.1002/jnr.23593 (2015). 25881750

[b23] Marquet P., Depeursinge C. & Magistretti P. J. Exploring neural cell dynamics with digital holographic microscopy. Annu Rev Biomed Eng. 15, 407–431, doi: 10.1146/annurev-bioeng-071812-152356 (2013). 23662777

[b24] Choi D. W., Maulucci-Gedde M. & Kriegstein A. R. Glutamate neurotoxicity in cortical cell culture. J Neurosci. 7, 357–368 (1987). 2880937 10.1523/JNEUROSCI.07-02-00357.1987 PMC6568898

[b25] Rothman S. M. The neurotoxicity of excitatory amino acids is produced by passive chloride influx. J Neurosci. 5, 1483–1489 (1985). 3925091 10.1523/JNEUROSCI.05-06-01483.1985 PMC6565259

[b26] Jourdain P. *et al.* Determination of transmembrane water fluxes in neurons elicited by glutamate ionotropic receptors and by the cotransporters KCC2 and NKCC1: a digital holographic microscopy study. J Neurosci. 31, 11846–11854, doi: 10.1523/JNEUROSCI.0286-11.2011 (2011). 21849545 PMC6623187

[b27] Pavillon N. *et al.* Early cell death detection with digital holographic microscopy. PloS one 7, e30912, doi: 10.1371/journal.pone.0030912 (2012). 22303471 PMC3269420

[b28] Allaman I. *et al.* Amyloid-beta aggregates cause alterations of astrocytic metabolic phenotype: impact on neuronal viability. J Neurosci. 30, 3326–3338, doi: 10.1523/JNEUROSCI.5098-09.2010 (2010). 20203192 PMC6634099

[b29] Belanger M. *et al.* Role of the glyoxalase system in astrocyte-mediated neuroprotection. J Neurosci. 31, 18338–18352, doi: 10.1523/JNEUROSCI.1249-11.2011 (2011). 22171037 PMC6623908

[b30] Marquet P. *et al.* Digital holographic microscopy: a noninvasive contrast imaging technique allowing quantitative visualization of living cells with subwavelength axial accuracy. Opt Lett. 30, 468–470 (2005). 15789705 10.1364/ol.30.000468

[b31] Rappaz B. *et al.* Measurement of the integral refractive index and dynamic cell morphometry of living cells with digital holographic microscopy. Opt Express. 13, 9361–9373 (2005). 19503137 10.1364/opex.13.009361

[b32] Rappaz B., Charriere F., Depeursinge C., Magistretti P. J. & Marquet P. Simultaneous cell morphometry and refractive index measurement with dual-wavelength digital holographic microscopy and dye-enhanced dispersion of perfusion medium. Opt Lett. 33, 744–746 (2008). 18382537 10.1364/ol.33.000744

[b33] Cuche E., Marquet P. & Depeursinge C. Simultaneous amplitude-contrast and quantitative phase-contrast microscopy by numerical reconstruction of Fresnel off-axis holograms. Appl Opt. 38, 6994–7001 (1999). 18324243 10.1364/ao.38.006994

[b34] Colomb T. *et al.* Numerical parametric lens for shifting, magnification, and complete aberration compensation in digital holographic microscopy. J Opt Soc Am A Opt Image Sci Vis. 23, 3177–3190 (2006). 17106474 10.1364/josaa.23.003177

[b35] Nicoletti I., Migliorati G., Pagliacci M. C., Grignani F. & Riccardi C. A rapid and simple method for measuring thymocyte apoptosis by propidium iodide staining and flow cytometry. J Immunol Methods. 139, 271–279 (1991). 1710634 10.1016/0022-1759(91)90198-o

[b36] Lecoeur H. Nuclear apoptosis detection by flow cytometry: influence of endogenous endonucleases.Exp Cell Res. 277, 1–14, doi: 10.1006/excr.2002.5537 (2002). 12061813

[b37] Trapnell C. *et al.* Differential gene and transcript expression analysis of RNA-seq experiments with TopHat and Cufflinks. Nat Protoc. 7, 562–578, doi: 10.1038/nprot.2012.016 (2012). 22383036 PMC3334321

[b38] Itoh Y. *et al.* Dichloroacetate effects on glucose and lactate oxidation by neurons and astroglia *in vitro* and on glucose utilization by brain *in vivo*. Proc Natl Acad Sci USA 100, 4879–4884, doi: 10.1073/pnas.0831078100 (2003). 12668764 PMC153649

[b39] Bouzier-Sore A. K., Voisin P., Canioni P., Magistretti P. J. & Pellerin L. Lactate is a preferential oxidative energy substrate over glucose for neurons in culture. J Cereb Blood Flow Metab. 23, 1298–1306, doi: 10.1097/01.WCB.0000091761.61714.25 (2003). 14600437

[b40] Halestrap A. P. The mitochondrial pyruvate carrier. Kinetics and specificity for substrates and inhibitors. Biochem J. 148, 85–96 (1975). 1156402 10.1042/bj1480085 PMC1165509

[b41] Halestrap A. P. & Denton R. M. Specific inhibition of pyruvate transport in rat liver mitochondria and human erythrocytes by alpha-cyano-4-hydroxycinnamate. Biochem J. 138, 313–316 (1974). 4822737 10.1042/bj1380313 PMC1166210

[b42] Poole R. C. & Halestrap A. P. Transport of lactate and other monocarboxylates across mammalian plasma membranes. Am J Physiol 264, C761–782 (1993). 8476015 10.1152/ajpcell.1993.264.4.C761

[b43] Khakh B. S. & North R. A. Neuromodulation by extracellular ATP and P2X receptors in the CNS. Neuron 76, 51–69, doi: 10.1016/j.neuron.2012.09.024 (2012). 23040806 PMC4064466

[b44] Fredholm B. B., AP I. J., Jacobson K. A., Klotz K. N. & Linden J. International Union of Pharmacology. XXV. Nomenclature and classification of adenosine receptors. Pharmacol Rev. 53, 527–552 (2001). 11734617 PMC9389454

[b45] Vogt A., Hormuzdi S. G. & Monyer H. Pannexin1 and Pannexin2 expression in the developing and mature rat brain. Brain Res Mol Brain Res. 141, 113–120, doi: 10.1016/j.molbrainres.2005.08.002 (2005). 16143426

[b46] Bao L., Locovei S. & Dahl G. Pannexin membrane channels are mechanosensitive conduits for ATP. FEBS letters 572, 65–68, doi: 10.1016/j.febslet.2004.07.009 (2004). 15304325

[b47] Fields R. D. & Burnstock G. Purinergic signalling in neuron-glia interactions. Nat Rev Neurosci. 7, 423–436, doi: 10.1038/nrn1928 (2006). 16715052 PMC2062484

[b48] Runden-Pran E., Tanso R., Haug F. M., Ottersen O. P. & Ring A. Neuroprotective effects of inhibiting N-methyl-D-aspartate receptors, P2X receptors and the mitogen-activated protein kinase cascade: a quantitative analysis in organotypical hippocampal slice cultures subjected to oxygen and glucose deprivation. Neuroscience 136, 795–810, doi: 10.1016/j.neuroscience.2005.08.069 (2005). 16344152

[b49] Engel T. *et al.* Seizure suppression and neuroprotection by targeting the purinergic P2X7 receptor during status epilepticus in mice. FASEB J. 26, 1616–1628, doi: 10.1096/fj.11-196089 (2012). 22198387

[b50] Erb L., Liao Z., Seye C. I. & Weisman G. A. P2 receptors: intracellular signaling. Pflugers Arch. 452, 552–562, doi: 10.1007/s00424-006-0069-2 (2006). 16586093

[b51] Arthur D. B., Georgi S., Akassoglou K. & Insel P. A. Inhibition of apoptosis by P2Y2 receptor activation: novel pathways for neuronal survival. J Neurosci. 26, 3798–3804, doi: 10.1523/JNEUROSCI.5338-05.2006 (2006). 16597733 PMC6674138

[b52] Brunet A., Datta S. R. & Greenberg M. E. Transcription-dependent and -independent control of neuronal survival by the PI3K-Akt signaling pathway. Curr Opin Neurobiol. 11, 297–305 (2001). 11399427 10.1016/s0959-4388(00)00211-7

[b53] Soundarapandian M. M., Zhong X., Peng L., Wu D. & Lu Y. Role of K(ATP) channels in protection against neuronal excitatory insults. J Neurochem. 103, 1721–1729, doi: 10.1111/j.1471-4159.2007.04963.x (2007). 17944875

[b54] Mirshamsi S. *et al.* Leptin and insulin stimulation of signalling pathways in arcuate nucleus neurones: PI3K dependent actin reorganization and KATP channel activation. BMC neuroscience 5, 54, doi: 10.1186/1471-2202-5-54 (2004). 15581426 PMC539348

[b55] Plum L. *et al.* Enhanced PIP3 signaling in POMC neurons causes KATP channel activation and leads to diet-sensitive obesity. J Clin Invest. 116, 1886–1901, doi: 10.1172/JCI27123 (2006). 16794735 PMC1481658

[b56] Olney J. W. *et al.* NMDA antagonist neurotoxicity: mechanism and prevention. Science 254, 1515–1518 (1991). 1835799 10.1126/science.1835799

[b57] Porras O. H., Loaiza A. & Barros L. F. Glutamate mediates acute glucose transport inhibition in hippocampal neurons. J Neurosci. 24, 9669–9673, doi: 10.1523/JNEUROSCI.1882-04.2004 (2004). 15509754 PMC6730152

[b58] Lee J. Y., Kim Y. H. & Koh J. Y. Protection by pyruvate against transient forebrain ischemia in rats. J Neurosci. 21, RC171 (2001). 11588201 10.1523/JNEUROSCI.21-20-j0002.2001 PMC6763857

[b59] Ryou M. G. *et al.* Pyruvate protects the brain against ischemia-reperfusion injury by activating the erythropoietin signaling pathway. Stroke 43, 1101–1107, doi: 10.1161/STROKEAHA.111.620088 (2012). 22282883 PMC3314723

[b60] Castillo X. *et al.* A probable dual mode of action for both L- and D-lactate neuroprotection in cerebral ischemia. J Cereb Blood Flow Metab. 35, 1561–1569, doi: 10.1038/jcbfm.2015.115 (2015). 26036941 PMC4640320

[b61] Xia J. *et al.* Neurons respond directly to mechanical deformation with pannexin-mediated ATP release and autostimulation of P2X7 receptors. J Physiol. 590, 2285–2304, doi: 10.1113/jphysiol.2012.227983 (2012). 22411013 PMC3424753

[b62] Kawamura M. Jr., Ruskin D. N. & Masino S. A. Metabolic autocrine regulation of neurons involves cooperation among pannexin hemichannels, adenosine receptors, and KATP channels. J Neurosci. 30, 3886–3895, doi: 10.1523/JNEUROSCI.0055-10.2010 (2010). 20237259 PMC2872120

[b63] Guthrie P. B. *et al.* ATP released from astrocytes mediates glial calcium waves. J Neurosci. **19**, 520–528 (1999). 9880572 10.1523/JNEUROSCI.19-02-00520.1999 PMC6782195

[b64] Iglesias R., Dahl G., Qiu F., Spray D. C. & Scemes E. Pannexin 1: the molecular substrate of astrocyte "hemichannels". J Neurosci. 29, 7092–7097, doi: 10.1523/JNEUROSCI.6062-08.2009 (2009). 19474335 PMC2733788

[b65] Fumagalli M. *et al.* Nucleotide-mediated calcium signaling in rat cortical astrocytes: Role of P2X and P2Y receptors. Glia 43, 218–203, doi: 10.1002/glia.10248 (2003). 12898701

[b66] Pedata F. *et al.* Purinergic signalling in brain ischemia. Neuropharmacology. doi: 10.1016/j.neuropharm.2015.11.007 (2015). 26581499

[b67] Ambrosio A. F., Malva J. O., Carvalho A. P. & Carvalho C. M. Inhibition of N-,P/Q- and other types of Ca^2+^ channels in rat hippocampal nerve terminals by the adenosine A1 receptor. Eur J Pharmacol. 340, 301–310 (1997). 9537827 10.1016/s0014-2999(97)01451-9

[b68] Deuchars S. A., Brooke R. E. & Deuchars J. Adenosine A1 receptors reduce release from excitatory but not inhibitory synaptic inputs onto lateral horn neurons. J Neurosci. 21, 6308–6320 (2001). 11487654 10.1523/JNEUROSCI.21-16-06308.2001 PMC6763129

[b69] Cunha R. A. Neuroprotection by adenosine in the brain: From A(1) receptor activation to A (2A) receptor blockade. Purinergic Signal. 1, 111–134, doi: 10.1007/s11302-005-0649-1 (2005). 18404497 PMC2096528

[b70] Nakata H., Yoshioka K., Kamiya T., Tsuga H. & Oyanagi K. Functions of heteromeric association between adenosine and P2Y receptors. J Mol Neurosci. 26, 233–238, doi: 10.1385/JMN:26:2-3:233 (2005). 16012196

[b71] Arbeloa J., Perez-Samartin A., Gottlieb M. & Matute C. P2X7 receptor blockade prevents ATP excitotoxicity in neurons and reduces brain damage after ischemia. Neurobiol Dis. 45, 954–961, doi: 10.1016/j.nbd.2011.12.014 (2012). 22186422

[b72] Di Virgilio F. Purines, purinergic receptors, and cancer. Cancer Res. 72, 5441–5447, doi: 10.1158/0008-5472.CAN-12-1600 (2012). 23090120

[b73] Yang J. *et al.* Lactate promotes plasticity gene expression by potentiating NMDA signaling in neurons. Proc Natl Acad Sci USA 111, 12228–12233, doi: 10.1073/pnas.1322912111 (2014). 25071212 PMC4143009

[b74] Mosienko V., Teschemacher A. G. & Kasparov S. Is L-lactate a novel signaling molecule in the brain? J Cereb Blood Flow Metab. 35, 1069–1075, doi: 10.1038/jcbfm.2015.77 (2015). 25920953 PMC4640281

[b75] Suzuki A. *et al.* Astrocyte-neuron lactate transport is required for long-term memory formation. Cell 144, 810–823, doi: 10.1016/j.cell.2011.02.018 (2011). 21376239 PMC3073831

[b76] Bliss T. M. *et al.* Dual-gene, dual-cell type therapy against an excitotoxic insult by bolstering neuroenergetics. J Neurosci. 24, 6202–6208, doi: 10.1523/JNEUROSCI.0805-04.2004 (2004). 15240812 PMC6729663

